# Translation of proteomic biomarkers into FDA approved cancer diagnostics: issues and challenges

**DOI:** 10.1186/1559-0275-10-13

**Published:** 2013-10-02

**Authors:** Anna K Füzéry, Joshua Levin, Maria M Chan, Daniel W Chan

**Affiliations:** 1 Department of Pathology, Johns Hopkins University, School of Medicine, Baltimore, MD 21287, USA; 2 Division of Immunology and Hematology Devices, Office of In Vitro Diagnostic Devices and Radiological Health, Center for Devices and Radiological Health, U.S. Food and Drug Administration, Silver Spring, MD, USA; 3 Center for Biomarker Discovery, Department of Pathology, Johns Hopkins Medical Institutions, Baltimore, MD 21231, USA; 4 Current address: Department of Pathology and Laboratory Medicine, Royal Alexandra Hospital, Diagnostic Treatment Center, Room 5047, Edmonton, AB T5H 3V9, Canada

**Keywords:** Proteomic biomarker, Analytical performance, Clinical performance, Food and drug administration

## Abstract

Tremendous efforts have been made over the past few decades to discover novel cancer biomarkers for use in clinical practice. However, a striking discrepancy exists between the effort directed toward biomarker discovery and the number of markers that make it into clinical practice. One of the confounding issues in translating a novel discovery into clinical practice is that quite often the scientists working on biomarker discovery have limited knowledge of the analytical, diagnostic, and regulatory requirements for a clinical assay. This review provides an introduction to such considerations with the aim of generating more extensive discussion for study design, assay performance, and regulatory approval in the process of translating new proteomic biomarkers from discovery into cancer diagnostics. We first describe the analytical requirements for a robust clinical biomarker assay, including concepts of precision, trueness, specificity and analytical interference, and carryover. We next introduce the clinical considerations of diagnostic accuracy, receiver operating characteristic analysis, positive and negative predictive values, and clinical utility. We finish the review by describing components of the FDA approval process for protein-based biomarkers, including classification of biomarker assays as medical devices, analytical and clinical performance requirements, and the approval process workflow. While we recognize that the road from biomarker discovery, validation, and regulatory approval to the translation into the clinical setting could be long and difficult, the reward for patients, clinicians and scientists could be rather significant.

## Introduction

A biomarker may be defined as a molecule that is objectively measured and evaluated as an indicator of normal biological processes, pathogenic processes, or pharmacologic responses to therapeutic intervention [1]. A tumor marker, in particular, is any molecule produced by a tumor or by the host in response to a cancer cell that is objectively measured and evaluated as an indicator of cancerous processes within the body [2]. Ideally, a tumor marker is detectable only in the presence of cancer but in practice the tumor markers of today lack such exquisite specificity. Current tumor markers may be grouped into a variety of categories including proteins, glycoproteins, oncofetal antigens, hormones, receptors, genetic markers, and RNA molecules. Moreover, tumor markers may be detected in sample matrices such as serum, plasma, whole blood, urine, and tissue.

Tremendous efforts have been made over the past few decades to discover novel cancer biomarkers for use in clinical practice. However, a striking discrepancy exists between the effort directed toward biomarker discovery and the number of markers that make it into clinical practice. Table 1 lists the handful of Food and Drug Administration (FDA) approved protein biomarkers in current clinical use. A number of excellent reviews, commentaries, and editorials have begun to address the source of this discrepancy and offer some insight into more successfully bridging the path from discovery to clinical diagnostics [3-9]. One of the confounding issues in translating a novel discovery into clinical practice is that quite often the scientists working on biomarker discovery have limited knowledge of the analytical, diagnostic, and regulatory requirements for a clinical assay [10]. This review provides an introduction to such considerations with the aim of generating more extensive discussion for study design, assay performance, and regulatory approval in the process of translating new proteomic biomarkers from discovery into cancer diagnostics.

**Table 1:** **List of FDA-approved protein tumor markers currently used in clinical practice**^**a**^

**Biomarker**	**Clinical use**	**Cancer type**	**Specimen**	**Methodology**	**Submission**	**Year first**	**Device**	**Product**
					**type**	**approved or**	**class**	**code**
						**cleared**		
Pro2PSA	Discriminating cancer from benign disease	Prostate	Serum	Immunoassay	PMA	2012	3	OYA
ROMA (HE4+CA-125)	Prediction of malignancy	Ovarian	Serum	Immunoassay	510(k)	2011	2	ONX
OVA1 (multiple proteins)	Prediction of malignancy	Ovarian	Serum	Immunoassay	510(k)	2009	2	ONX
HE4	Monitoring recurrence or progression of disease	Ovarian	Serum	Immunoassay	510(k)	2008	2	OIU
Fibrin/ fibrinogen degradation product (DR-70)	Monitoring progression of disease	Colorectal	Serum	Immunoassay	510(k)	2008	2	NTY
AFP-L3%	Risk assessment for development of disease	Hepatocellular	Serum	HPLC, microfluidic capillary electrophoresis	510(k)	2005	2	NSF
Circulating Tumor Cells (EpCAM, CD45, cytokeratins 8, 18+, 19+)	Prediction of cancer progression and survival	Breast	Whole blood	Immunomagnetic capture/ immune-fluorescence	510(k)	2005	2	NQI
p63 protein	Aid in differential diagnosis	Prostate	FFPE tissue	Immunohistochemistry	510(k)	2005	1	NTR
c-Kit	Detection of tumors, aid in selection of patients	Gastrointestinal stromal tumors	FFPE tissue	Immunohistochemistry	PMA	2004	3	NKF
CA19-9	Monitoring disease status	Pancreatic	Serum, plasma	Immunoassay	510(k)	2002	2	NIG
Estrogen receptor (ER)	Prognosis, response to therapy	Breast	FFPE tissue	Immunohistochemistry	510(k)	1999	2	MYA
Progesterone receptor (PR)	Prognosis, response to therapy	Breast	FFPE tissue	Immunohistochemistry	510(k)	1999	2	MXZ
HER-2/neu	Assessment for therapy	Breast	FFPE tissue	Immunohistochemistry	PMA	1998	3	MVC
CA-125	Monitoring disease progression, response to therapy	Ovarian	Serum, plasma	Immunoassay	510(k)	1997	2	LTK
CA15-3	Monitoring disease response to therapy	Breast	Serum, plasma	Immunoassay	510(k)	1997	2	MOI
CA27.29	Monitoring disease response to therapy	Breast	Serum	Immunoassay	510(k)	1997	2	MOI
Free PSA	Discriminating cancer from benign disease	Prostate	Serum	Immunoassay	PMA	1997	3	MTG
Thyroglobulin	Aid in monitoring	Thyroid	Serum, plasma	Immunoassay	510(k)	1997	2	MSW
Nuclear Mitotic Apparatus protein (NuMA, NMP22)	Diagnosis and monitoring of disease (professional and home use)	Bladder	Urine	Lateral flow immunoassay	PMA	1996	3	NAH
Alpha-fetoprotein (AFP)^b^	Management of cancer	Testicular	Serum, plasma, amniotic fluid^b^	Immunoassay	PMA	1992	3	LOK
Total PSA	Prostate cancer diagnosis and monitoring	Prostate	Serum	Immunoassay	PMA	1986	2	LTJ, MTF
Carcino-embryonic antigen	Aid in management and prognosis	Not specified	Serum, plasma	Immunoassay	510(k)	1985	2	DHX
Human hemoglobin (fecal occult blood)	Detection of fecal occult blood (home use)	Colorectal	Feces	Lateral flow immunoassay	510(k) – CLIA waived	1976	2	KHE

^a^ While hCG is commonly used as a tumor marker, it has not been cleared/approved for this application by the FDA.

^b^ AFP is a Class III analyte because of its non-cancer intended use (aid in prenatal diagnosis of birth defects).

## Biomarker discovery

A formal structure to guide the process of biomarker development was proposed by Pepe and colleagues [11] and adopted by the National Cancer Institute Early Detection Research Network (EDRN). The five phases of biomarker development include (1) preclinical exploratory (2) clinical assay validation (3) retrospective longitudinal (4) prospective screening and (5) cancer control. The goal of preclinical exploratory studies is to identify one or more promising tumor markers which can then be further developed in subsequent stages of the pipeline. Currently, one of two common approaches is taken to identify a new potential tumor marker: unbiased high throughput discovery or targeted discovery. While unbiased high throughput discovery is frequently used, targeted discovery is now being promoted as the preferred approach by many groups [6,9,12]. The key advantage of the latter approach is that defining an intended use for the tumor marker at the early stages of the discovery process allows better control of the variables (other than the cancer itself) that may influence measured levels of the marker during the discovery process.

Regardless of which approach is taken, a careful study design is essential to success. Some of the issues to be considered when deciding on a discovery strategy include the number of samples to analyze, inclusion/exclusion criteria for the samples, collection and handling requirements, downstream effects of sample processing, limitations of the analytical methodology(ies), appropriate statistical analysis of the acquired data, and validation of the findings in independent datasets and by independent investigators. Moreover, complete and transparent reporting of results is also necessary so that other investigators can assess the soundness of the study. For an in-depth discussion of these and other considerations, the reader is referred to several excellent published reviews [4,6,8,10,13-16].

## Assay development: analytical performance

### General considerations

Once a promising tumor marker candidate is identified, the next step is to develop an assay with suitable analytical performance for diagnostic accuracy studies and for eventual use of the assay in routine clinical practice. One must keep in mind that proteomic technologies used in biomarker discovery are generally not transferable to clinical laboratories owing to their high complexity, relatively low throughput, and their analytical performance characteristics. Therefore, the evaluation of alternate methodologies early on is highly advisable. The OVA1 test for ovarian cancer (Vermillion, Inc), for example, was initially developed on the SELDI platform because this platform had been used to discover the five proteomic biomarkers included in the test. However, despite significant efforts the precision of the test could not be increased to clinically acceptable levels (see below for a discussion on the levels of precision that are clinically acceptable). The SELDI platform was therefore abandoned and the test was then successfully implemented using immunoassays [17].

A plethora of guidelines exist on how to establish the performance of an assay [18-28] and it is not always evident which guideline best applies to a particular assay. A general rule of thumb is that the intended use and the targeted country’s regulatory requirements should both be taken into consideration when determining the stringency of performance assessment. In order to develop an analytically robust tumor marker assay, however, at least the following parameters should be evaluated: precision, trueness, limit of detection, limit of quantitation, linearity and working range, specificity, carryover, and analyte stability. Considerations for some of these parameters will be described in the following sections; for additional information the reader is referred to the many method validation guidelines available in literature.

### Precision

Precision refers to the closeness of agreement between a series of measurements obtained for the same sample under a specified set of conditions. Precision evaluates random error and may be identified as within-runbetween-runwithin-day, between-day, or within-laboratory [21]. Of these, within-run and within-laboratory precision are usually the first requirements for a good assay. Within-run precision, often called repeatability, applies to successive measurements performed under identical conditions; within-laboratory precision, often called reproducibility, applies to measurements performed under a variety of conditions that includes different days of the week or month, and differences in operators, calibrators, reagent lots, ambient temperature, and so forth [21,29]. Precision is quantitatively expressed in terms of the standard deviation (SD), variance, or coefficient of variation (CV) of a series of measurements. Precision is often a function of the analyte concentration, with small concentrations resulting in poorer precision (i.e. larger SD, variance, and CV) than high concentrations.

When a new tumor marker assay is developed, precision should be evaluated across the entire reportable range of the assay. Particular care should be taken that the precision is assessed at the medical decision points of relevance to the intended clinical application of the tumor marker [21]. Patient pooled samples are the most ideal for evaluation, but if these are not available then test materials that stimulate the characteristics of appropriate clinical samples may be used [21]. Since cancer patients are often monitored over long periods of time, evaluation of the long-term stability of the assay is of particular importance.

Currently, the decision as to what constitutes an acceptable level of precision is based on one or more factors including intended clinical application, biological variation, regulatory guidelines, medical opinions, and guidelines from professional groups [30]. Biological variation refers to the random fluctuation of an analyte concentration around a homeostatic setpoint [31]. This fluctuation may occur within a single patient (intraindividual variation) and/or across multiple individuals (interindividual variation). Because several tumor markers show biological variation [32], precision criteria derived in this way appear to be a feasible option. However, there are a number of practical limitations to this approach including the time required to accumulate sufficient data and the difference between healthy and malignant states [32]. In the absence of biological variation data, performance criteria for already established tumor markers and recommendations from professional groups may be the most reasonable starting points. The reader is referred to a number of excellent articles [31-33] for more details.

Ultimately, whether a degree of precision is acceptable or not will be influenced to a large extent by the intended clinical use of the test. Decisions based on a single tumor marker result, for example diagnosis or prognosis, will require high within-run precision while decisions based on time-related changes, for example in the monitoring of therapy, will require high within-laboratory precision.

### Trueness

Trueness refers to the closeness of agreement between the average value obtained from a large series of test results and a true or an accepted reference value [34]. Trueness evaluates systematic error and is quantitatively expressed in terms of bias. Trueness is inversely related to bias i.e. the greater the bias, the greater the discrepancy between the average measured value and the true value. Constant bias yields results that differ from the true value by a fixed amount; proportional bias yields results differing by an amount that is proportional to the concentration of the measurand. The presence of bias may not appear important during the translation of a biomarker into clinical practice. However, as multiple assays eventually become available for the marker, a bias in any one assay will complicate the diagnosis and long-term monitoring of patients [33]. While standardization efforts can address such problems, they are generally very challenging and time-consuming. For this reason, it is advisable to evaluate and minimize bias as much as possible during the translational stage.

Bias is strongly influenced by the specificities of the antibodies used in an immunoassay. Many tumor markers exist in biological fluid as a family of isoforms and antibodies from different assays may recognize distinct subsets of this family. Human choriogonadotropin (hCG), for example, exists in the intact form, as the free α- and β-subunits, as a nicked form of the β-subunit, as the core fragment, and as hypo- and hyperglycosylated forms [35]. However, not all forms exist in every type of body fluid and the relative proportion of isoforms may vary among health and disease states [35-39]. The relative specificities of the antibodies used in an hCG assay are an important determinant of which hCG forms are measured and, therefore, of the reported hCG value [35,40]. (Please note that hCG has not been cleared/approved by FDA as a cancer diagnostics.) Another example is prostate specific antigen (PSA). PSA exists in multiple isoforms. The predominant form in blood is PSA-ACT (PSA bound to α_1_-antichymotrypsin) but other forms are also of relevance including free PSA [41]. Current assays detect both free PSA and PSA-ACT but differences exist as to the relative response elicited by each form [42,43]. Equimolar assays generate identical signals for equal molar concentrations of free PSA and PSA-ACT while skewed assays respond differently to the two forms. As these examples illustrate, a clear understanding of what forms are being measured is essential for the assessment of assay bias and for meaningful result interpretation. As a result, when developing a new assay it is important to thoroughly characterize the tumor marker of interest and to carefully evaluate and report the specificities of the reagent antibodies.

In addition to reagent antibody specificities, assay calibration may also produce bias. Calibration refers to the process whereby a relationship between instrument signal and known amounts of analyte is established; a sample with a known amount of analyte is called a calibrator [29]. Ideally, the composition of the calibrator should closely resemble the patient specimens that will be analyzed in routine clinical practice i.e. it should be commutable. Moreover, if calibrators contain more than one form of the analyte then the relative proportion of each form needs to be accurately defined. These criteria are often tricky to fulfill for tumor markers, in part because the structural heterogeneity of a new marker may not be completely understood at the time of assay development and in part because analytical techniques may not be of sufficient quality to allow preparation of the desired calibrator materials. Nevertheless, a tumor marker assay with imperfect calibration may still be used successfully to manage cancer patients if the effects of the calibration on bias are kept in mind [44]. hCG is one illustrative example; even though cancer-related applications of hCG have existed for several decades, standards of high purity and homogeneity for six of the most important hCG isoforms have only recently become available [45].

### Limit of detection and limit of quantitation

The limit of detection (LoD) represents the lowest amount of an analyte that can be reliably distinguished from zero. Terms used interchangeably with LoD, including lower limit of detection, minimum detectable concentration, analytical sensitivity, and biological limit of detection, have led to confusion regarding how to evaluate this performance indicator. One procedure involves repeated measurement of a zero standard (i.e. a sample lacking the analyte of interest) within a single run. After determining the mean and SD of the results, the LoD is then calculated from the mean plus two (or three) SDs [18,25,46]. Although this is a frequently used approach, it is often overly optimistic and produces LoD values that cannot be repeated during the day-to-day operations of a laboratory. A more appropriate estimate of the LoD may be obtained by an alternative approach [47,48]. First, the limit of blank (LoB) is determined using repeated measurements of a zero standard and the formula of “LoB equals the mean of the measurements plus 1.65 times the SD”. The LoD is subsequently determined from a combination of the LoB and repeated measurements of a sample with a low concentration of analyte.

While a low LoD is desirable for many clinical applications of tumor markers, including early detection and monitoring of recurrence, it is not sufficient for clinical use. As discussed earlier, the assay also needs to have acceptable precision and bias at these low levels of analyte. Otherwise, any result near the LoD may have so much uncertainty associated with it that it no longer allows confident clinical interpretation and decision making. A performance indicator that incorporates these requirements is the limit of quantitation (LoQ), the lowest concentration at which the analyte can not only be reliably distinguished from zero but also meets certain specifications for bias and precision [46,48]. Other terms that have been used interchangeably with LoQ include lower limit of determination, lower end of the measuring range, lower limit of quantitation, and limit of quantification [48]. In most instances LoQ exceeds LoD but it’s possible for the two quantities to be equal. Considerations for establishing the LoQ of an assay are similar to those for establishing the LoD.

### Specificity and analytical interference

Analytical interference may be defined as the effect of a substance in a sample that alters the correct value of the result [49]. In today’s clinical laboratory, protein tumor markers in biological fluid are generally measured by two-site, non-competitive immunoassays (“sandwich immunoassays”) due to the good analytical characteristics, wide availability, and highly automatable nature of this technique [14]. However, this technique also has a number of well-known limitations that must be considered during assay development and validation. One limitation, as discussed in the section on trueness, is that the reagent antibodies are frequently not completely specific for a single molecular species. Cross-reactivity may occur with other isoforms of the marker, with more distantly related molecules, and even with unrelated molecular species.

**Figure 1:**
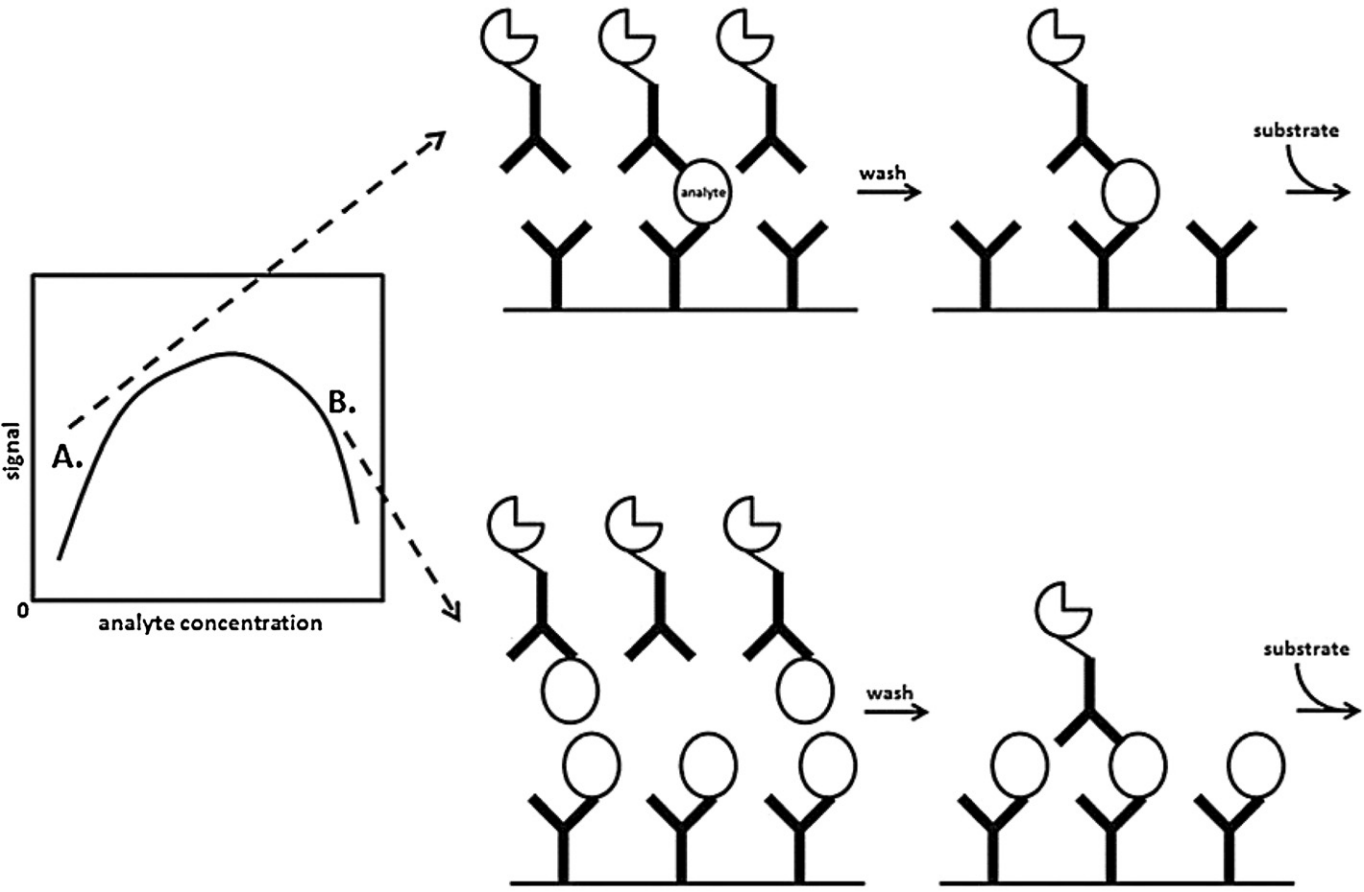
**Illustration of the hook effect.** This phenomenon arises because high concentrations of analyte saturate all antigen binding sites on the capture and label reagent antibodies and thereby interfere with sandwich-formation. A subsequent wash step removes all species not bound to the capture antibody (including analyte-label antibody complexes) and leads to a lower-than-expected signal during detection. **(A)** The analyte concentration is low relative to the number of available antibody binding sites. A hook effect does not occur. **(B)** The analyte concentration is high relative to the number of available antibody binding sites. The hook effect leads to falsely low signal.

The hook effect is another important limitation of sandwich immunoassays [50]. The hook effect refers to the phenomenon where a sample with a high analyte concentration gives a signal that is much lower than the theoretically expected value. This phenomenon arises because high concentrations of analyte saturate all antigen binding sites on the capture and label reagent antibodies and thereby interfere with sandwich-formation (Figure 1). A subsequent wash step removes all species not bound to the capture antibody (including analyte-label antibody complexes) and leads to a lower-than-expected signal during detection. The hook effect is of particular concern to tumor marker assays because the concentration of a tumor marker may range over several orders of magnitude and, in some cases, may even exceed millions of units per liter [51-54]. Case reports of the hook effect causing a falsely low tumor marker result and thereby leading to adverse patient outcomes are not uncommon. O’Reilly and Rustin related the case of a falsely low hCG measurement that led to an unnecessary hysterectomy for a 43-year-old woman and also delayed the diagnosis and treatment of her metastatic choriocarcinoma [54]. In another report, Jassam and colleagues described a two-month-old infant with a liver tumor whose diagnosis and subsequent management was changed from hepatic haemangioendothelioma to hepatoblastoma after discovery of a falsely low alpha-fetoprotein (AFP) measurement [52]. To avoid such errors as best possible, the National Academy of Clinical Biochemistry recommends that every testing laboratory have defined protocols in place for identifying specimens that have “hooked” [33]. Dilution and re-measurement of a specimen is one way to identify a hook effect but financial and time considerations prevent this from being applied to every specimen that arrives in a laboratory. In some cases, strong clinical suspicion of an erroneous result will suggest that the dilution protocol be applied, but in many cases the erroneous result may go unnoticed. For this reason, it is imperative that the potential for hook effect is minimized before the assay is implemented in the laboratory i.e. during the development phase. Other common interferences that have been documented for sandwich immunoassays include anti-reagent antibodies which are found in patient samples (e.g. human anti-mouse antibodies and rheumatoid factor) and nonspecific interferences [55-58]. However, one should keep in mind that these are not the only interferences that may occur, and a careful study with samples from the intended use population should be included as part of the assay validation.

### Carryover

Carryover may be defined as the process by which materials, such as parts of a specimen or reaction reagent, are unintentionally transferred from one reaction mixture into another [59]. Carryover may produce erroneous results that go undetected and in such cases it jeopardizes the optimal clinical management of patients. In automated assays, the risk of introducing clinically significant errors due to carryover is particularly high if a low concentration sample is analyzed immediately after an extremely high concentration sample [60]. Examples of tumor markers for which such carryover has been documented include AFP, hCG, and carcinoembryonic antigen (CEA) [51]; this is consistent with the observation made earlier that these markers may range over several orders of magnitude and even exceed millions of units per liter in some cases. Laboratories often take steps to limit the downstream effects of any recognized carryover, for example by retesting a sample that follows directly after a high concentration sample [51]. However carryover may sometimes be missed because of concomitant analytical problems such as the hook effect. For example, in the case of the two-month-old baby described earlier it is unlikely that any concerns over carryover arose for the sample with an AFP concentration of ~1 844 000 kU/L because the result was falsely lowered to 452 kU/L by the hook effect [52]. Another instance where significant carryover may be missed is when the tumor marker that carries over is not actually measured for the first of two consecutive specimens [61]. To minimize the occurrence of such scenarios, it is imperative that carryover be minimized during assay development. The use of disposable pipette tips and sample cups, careful selection of sample and reagent probe materials, and optimization of probe design and system wash procedures are just a few of the strategies currently used to minimize carryover in automated test systems [60,62].

## Assay development: clinical performance

### General considerations

Robust analytical performance is an essential but insufficient prerequisite for the successful clinical deployment of a novel tumor marker. Diamandis provided accounts of initially promising tumor markers that failed to make it into clinical practice because of errors made during validation of the markers’ clinical performance [4]. Ioannidis related similar stories and classified biomarker failures into four common types: type A (clinical reversal), type B (validation failure), type C (nonoptimized clinical translation), and type D (promotion despite nonpromising evidence) [5]. Of these four failure types, only one is related to the analytical performance of the tumor marker with the other three being due to insufficient clinical validation. Clinical validation of a tumor marker is complex, time-consuming, and expensive; careful planning at every stage is therefore essential to avoid a waste of resources.

### Intended use

It is extremely important to realize that the clinical performance of a tumor marker will vary across clinical contexts, disease spectra, and patient subgroups [63]. Therefore, a clear definition of the intended clinical use of the tumor marker is essential for designing an appropriate validation study. Parameters that should be defined include the disease of interest, the decision-making point along the disease progression path, the patient population for which the biomarker is intended, and the costs of true positive, true negative, false positive, and false negative results [9]. It is also important to recognize that a test is generally part of a management pathway. Thus, for successful integration into clinical practice a test must either improve on the diagnostic accuracy of the entire pathway or it must provide an alternate tangible benefit such as reduced monetary cost [64]. Although defining an intended use is often the most challenging part of a project, close collaboration with clinical staff goes a long way in facilitating this process.

### Clinical sensitivity and specificity

Clinical sensitivity and specificity are both measures of the intrinsic diagnostic accuracy of a test. Clinical sensitivity is the ability of the test to correctly identify those patients with the disease of interest; clinical specificity is the ability to correctly identify those patients without the disease of interest [65]. An ideal assay demonstrates clinical sensitivity and specificity of 100% but this is never achieved in practice because an increase in sensitivity is only gained at the expensive of specificity and vice versa. The decision to maximize sensitivity, specificity, or both depends on many factors including the general course of the disease, the consequences of early versus late diagnosis, the consequences of a false positive or negative result, and, to some extent, the financial costs associated with testing and subsequent patient care.

Several hierarchical models have been proposed to evaluate the diagnostic performance of a test [66,67]. Zhou and colleagues, for example, proposed three phases: the exploratory, the challenge, and the advanced phase [67]. The exploratory phase evaluates the discriminatory power of the test by comparing patients with classical overt disease to healthy controls. Such studies use small sample sizes, and also tend to overestimate the clinical performance of the test due to the inclusion of easy-to-diagnose patients in the study population. Nevertheless, the exploratory phase is critical for assessing whether a test has any discriminatory power and whether it is worthwhile to pursue it any further. The exploratory phase is followed by the challenge phase. This phase evaluates the discriminatory power of the test with respect to challenging patient populations with and without the disease of interest. Patients included in the study may have early or subtle disease, may have other comorbidities, or may have an unrelated disease localized to the same anatomic location. These studies generally underestimate the diagnostic accuracy of the test but may help to identify ways to improve the test before further evaluation. The last phase of evaluation, called the advanced phase, determines the test’s clinical performance in a study group that is representative of the target patient population for the test. Such studies are prospective, randomized, always require large numbers of patients, and are often multi-institutional in nature. Although such studies require large financial and time investments, they provide critical proof of the test’s diagnostic accuracy for prospective users.

The proper study design for each phase may be quite challenging. Careful consideration must be given to many issues including: (1) the availability (choice) of a technique for determining the true disease status of each individual, (2) identifying appropriate individuals to be included in the target populations, (3) the size of the study populations, and (3) appropriate and standardized interpretation of results from the test under evaluation. An additional confounding issue is that tumor markers may be used in applications other than diagnosis, and in these situations it may be unclear how to define the target condition or clinical sensitivity and specificity. For an in-depth discussion of these, and additional considerations, the readers are referred to several excellent articles [4,63,66-71].

### ROC analysis

**Figure 2:**
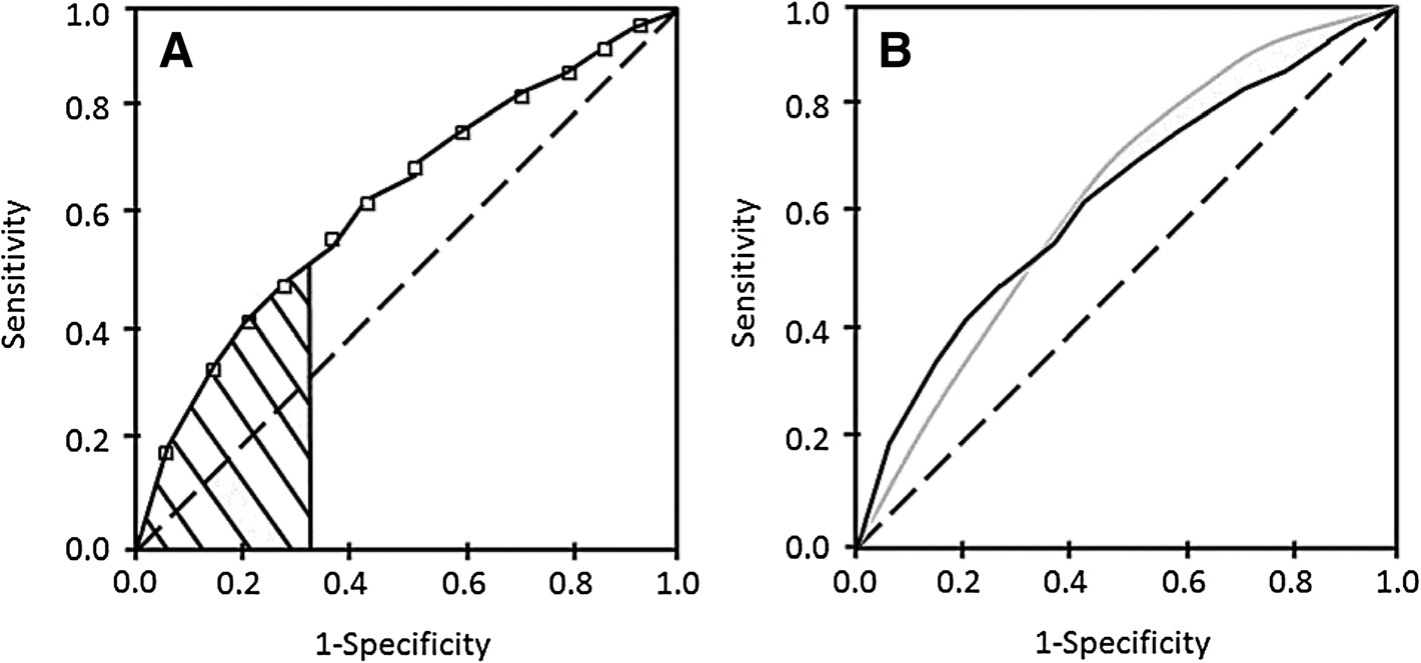
**Hypothetical ROC curves. (A)** A hypothetical non-parametric ROC plot. Each open square corresponds to the sensitivity and (1 minus specificity) values obtained for a particular decision threshold. The dashed diagonal line corresponds to the random chance line. The hashed region corresponds to the PAUC for the range of specificities between 68% and 100%. **(B)** Two hypothetical ROC curves with identical AUCs but different performances over the range of thresholds.

Clinical sensitivity and specificity depend intimately on the decision threshold used for a test. A decision threshold is a predetermined, fixed value of an analyte that, when exceeded, indicates that a critical decision needs to be made with respect to the patient’s care. Receiver operating characteristic (ROC) analysis is a powerful tool to evaluate diagnostic test performance and is invaluable in the selection of a decision threshold that is appropriate for the intended clinical use of the test. A hypothetical ROC curve is shown in Figure 2A. Popular ways of selecting an optimal threshold include finding the point on the curve closest to the coordinate (x = 0, y = 1), and calculation of the Youden index [72-74]. Both of these methods give equal weight to sensitivity and specificity but fail to consider disease prevalence and the financial, emotional, and ethical costs of misdiagnoses. Although the latter considerations are very important in clinical practice, they are difficult to quantify and, as a result, are rarely incorporated into ROC analyses.

One property of ROC curves that is extremely useful in the clinical validation of a tumor marker is the area under the curve (AUC). This parameter is a combined measure of the sensitivity and specificity of a test over all threshold values, and is thus a useful indicator of the marker’s overall diagnostic performance [73,75]. A higher AUC indicates better performance; thus an ideal assay (100% sensitivity and specificity) will have an AUC of 1.0 while an assay with no power to discriminate diseased from healthy individuals will have an AUC of 0.5. In certain cases, however, the AUC will be too global of a summary measure and will not accurately reflect the performance of the assay for the intended clinical application. A more meaningful way to evaluate the diagnostic accuracy in such cases is to calculate the partial area under the curve (PAUC) i.e. the AUC over a selected range of threshold values that covers the specificities of clinical interest (Figure 2A) [75,76].

In addition to evaluating a single assay, ROC curves may also be used to compare multiple assays or different generations of a single assay. In such instances, a visual examination of the ROC plot should always be done concomitantly with the assessment of quantitative parameters (e.g. AUC, PAUC) because assays can have identical AUCs or PAUCs and still perform differently over the range of threshold values examined (Figure 2B) [72,75,76].

While ROC analysis is very helpful in assessing the diagnostic performance of an assay, users need to be mindful of its limitations. For an in-depth discussion of common misuses of ROC curves, the reader is referred to a report by Obuchowski and colleagues [77].

### Positive and negative predictive values

When evaluating the diagnostic performance of an assay, the prevalence of the disease in the target patient population also needs to be taken into account. The positive predictive value (PPV) and negative predictive value (NPV) combine prevalence with the test’s clinical sensitivity and specificity. The PPV provides the likelihood that, given a positive test result the patient actually has the disease in question. Similarly, the NPV provides the likelihood that, given a negative test result, the patient does not have the disease. A test may have very high diagnostic accuracy and still have a low PPV or NPV, thereby limiting the test’s adoption into clinical practice. Cancer antigen 125 (CA-125) clearly illustrates this problem; the sensitivity and specificity of CA-125 for ovarian cancer could be set close to ~100%, but, owing to the low prevalence of ovarian cancer, the PPV of CA-125 for ovarian cancer is only 4%. Thus, out of every 100 positive test results only 4 will signal the presence of real disease [78]. This problem exists not only for CA-125 but for most other tumor markers and it is one reason for the current paucity in cancer screening assays. Therefore, some attempt should always be made to assess the PPV and NPV of a test during the evaluation of its clinical performance.

### Clinical utility

Clinical utility is the degree to which the use of a test leads to improved patient outcomes and cost-effective care [63]. This concept encompasses not only the intended use of a test but also the benefits and harms of the test to a single patient and to society as a whole [79]. Although a demonstration of clinical utility is not necessary for FDA clearance or approval, more and more decision makers demand evidence of clinical utility before electing to reimburse the cost of a test or intervention [16,69,80].

Clinical utility is best assessed through randomized controlled trials as these are the least prone to bias. However, such studies are often costly, require very large sample sizes, and have ethical challenges and therefore are not always feasible [69,79]. Modeling studies may be a feasible alternative but care must be taken to ensure that input variables are supported by strong evidence and that any other assumptions are also valid for the case at hand [16,79]. A third approach that is sometimes used is a before-after study. Here, data is obtained for a group before the introduction of a test and it is compared with data obtained after the introduction of the test. A major drawback of this type of study is that it fails to consider other changes that may have occurred during the study period. Thus, the influence of the new test on the measured outcomes remains somewhat uncertain [79].

## Assay development: additional considerations

In addition to the factors discussed in the previous sections, assay development should consider a number of practical issues that may affect acceptance of the assay by the healthcare community. There is increasing pressure on laboratories to report results rapidly and efficiently. Studies indicate that lengthy turnaround times, i.e. the time between ordering a lab and receiving the result, lead to dissatisfaction of physicians, nurses, and patients [81-83]. It is also quite likely that lengthy turnaround times reduce physician efficiency and increase the length of hospital stays [81,84]. For this reason, it is advisable to minimize turnaround times through computerized physician order entry, optimized laboratory workflows, computerized result verification and reporting, and similar efforts [81]. Importantly, reducing the length of time required for test setup and completion can also contribute to reduced turnaround times. Therefore, whenever possible, assay development should not only concentrate on analytical and clinical performance but also on reducing the time required to perform the assay.

Financial cost of the assay is another factor that should be minimized during development and validation. Owing to the poor state of the economy, governmental spending on healthcare is being reduced in many countries. In contrast, the aging population of the world is increasing and will require an ever increasing number of laboratory tests, including tumor markers. As a result, more and more insurers are including cost-effectiveness in their decision to pay or not pay for a test [78]. For this reason, every effort should be made during the assay development to minimize the costs associated with performing the test. In addition, assay developers might consider performing cost-effectiveness studies to determine if their assay will make economic sense from the payer’s and society’s perspective [78]. For a more detailed discussion of financial considerations, the reader is referred to an excellent review by Scott [78].

## FDA approval process for protein-based biomarkers

### Classification of protein-based biomarker assays as medical devices

Protein-based biomarker assays used for diagnosis, monitoring and treating disease are considered by FDA to be medical devices and follow the same regulatory standards as other types of medical devices. Most protein-based biomarker assays are regulated by the Office of In Vitro Diagnostics and Radiological Health in FDA’s Center for Devices and Radiological Health (CDRH). A few diagnostic devices, primarily those dealing with HIV or blood banking analytes, as well as human leukocyte antigen analysis, are regulated by the Center for Biologics Evaluation and Research.

Medical devices are divided by FDA into three different classes, depending on the intended use of the device and the risk to the patient that arises when the device provides incorrect results, e.g. false positives or false negatives. The intended use describes to what extent the device itself will be used to make diagnostic and/or therapeutic decisions. Class III devices, devices that pose significant risk to the patient, require premarket approval by FDA (submission of a premarket approval application, or PMA), including a pre-approval inspection of the manufacturing site. The standard for premarket approval is that the sponsor (the firm submitting the premarket approval application) must demonstrate, through analytical and clinical performance studies, that the device is safe and effective for use in patient care. Only PMAs are “approved”.

Moderate risk (class II) devices are reviewed by FDA through the premarket notification [otherwise known as the 510(k)] pathway. The concept of premarket notification is that the sponsor informs FDA that they will be introducing the class II device into the market in 90 days, unless FDA raises any concerns before the 90 days are up. The appropriate terminology is that for the 510(k) pathway FDA “clears” the class II device for marketing. The regulatory standard for 510(k) review is that the sponsor must demonstrate that the device is “substantially equivalent” to a predicate device. A predicate device is a class II device that has been previously cleared by FDA, or was marketed prior to the introduction of the 510(k) program in 1976. In principle, a predicate device (possibly several generations back) originally demonstrated that it is safe and effective when cleared. The new device has only to demonstrate that its performance is substantially equivalent to the performance of the predicate, implying therefore that it is at least as safe and effective as the predicate device. The sponsor is required to list similarities and differences between the new device and the predicate device and to demonstrate equivalent performance between the two devices.

Demonstrating substantial equivalence to a predicate device is generally a lower burden of proof for a sponsor than demonstrating for the first time that the device is safe and effective. Depending on the analyte, a 510(k) application does not always require a clinical study; instead, a study comparing the results obtained with the predicate device with those obtained the new device may suffice to demonstrate substantial equivalence. For devices that are moderate risk, but where no appropriate predicate device exists, CDRH permits 510(k) clearance through a special process known as the “De Novo” pathway. Sponsors utilizing the De Novo pathway are required to demonstrate the device is safe and effective, but unlike Class III devices, a premarket inspection is not required. The advantage of the De Novo pathway for manufacturers is that it permits novel biomarker tests to reach the market more quickly, and with less expense and a lower postmarket burden on reporting changes to the test. If a sponsor is considering utilizing the De Novo pathway, the sponsor should engage with FDA through the presubmission process to craft an intended use statement that will allow the biomarker test to be regulated as a Class II device. Once FDA and the sponsor have agreed on an intended use, the biomarker test can be reviewed under the 510(k) program and FDA will draft a new regulation for the device. Many of the biomarker tests listed in Table 1 were originally cleared through the De Novo pathway.

The lowest risk device, Class I device, does not require a premarket submission to FDA; however, the firm manufacturing the Class I device is still required to register & list the device with the agency, and is also responsible for most aspects of the medical device quality system, including the reporting of adverse events and product recalls.

### Clinical and analytical requirements for biomarker performance

The intended use of a device is paramount, and all clinical and analytical requirements for biomarker performance derive from the intended use. These requirements can vary substantially. For example, biomarkers used in the diagnosis of neoplastic disease are generally considered Class III devices, while biomarkers used for monitoring or prognosis of cancer are generally Class II devices. The harm done to the patient from incorrectly identifying prognosis of disease is generally considered to be lower than the harm done by incorrect diagnosis. Immunohistochemistry protein biomarker assays are handled as a special case; the risk factors that determine whether an immunohistochemistry method is class I, II, or III is clearly delineated as part of the regulation 21 CFR 864.1860, “Immunohistochemistry reagents and kits”.

For protein biomarker assays, validation and standardization of pre-analytical steps are critical for assay reproducibility. For example, a multi-site assessment demonstrated that inter-laboratory differences, due to differences in pre-analytical methods, were the most significant source of variation in the reproducibility of a multiple reaction monitoring-based mass spectrometry biomarker assay [85]. Another critically important consideration for protein biomarker assays is for the assay developer to define the algorithm and clinical cutoff, if applicable. FDA expects that the sponsor will provide feasibility data, normal range studies, and/or training set data to demonstrate how the algorithm or cutoff was determined. Clinical study samples used to define an algorithm cannot be re-used to validate that same algorithm. Once the algorithm has been locked down, fresh samples should be used for the clinical validation study avoid introducing a type of verification bias into the study.

Analytical study requirements for biomarker performance are similar between class II and class III devices. Performance of the assay around the clinical decision point is the most important feature of the test’s performance. This decision point may be the dividing line between a “positive” or “negative” result for a qualitative assay, or may encompass the entire measuring range of a quantitative assay. Sponsors should pre-define the acceptance criteria prior to performing the pivotal analytical studies, and should justify any criteria that are unexpected. Generally, analytical validation studies usually include the following components: precision, linearity, limit of detection, limit of quantitation, analytical specificity, and matrix comparisons. The stability of the device, including sample stability upon storage, preparation, etc., must be demonstrated. The sponsor also needs to verify the as stability of any calibrators and/or controls that are supplied with the kit or as stand-alone accessories.

Algorithms and software used to determine a result also are reviewed by FDA. When software algorithms are used to generate a single result from the results of multiple tests, these algorithms are considered devices in and of themselves. OVA1 is an example of such a device.

Sponsors submitting 510(k) applications can use FDA’s 510(k) database to download the decision summary of the predicate and additional related devices; this decision summary is drafted by FDA to summarize the analytical and clinical studies used in the clearance of the device. Predicate device decision summaries are very helpful to sponsors in planning studies for class II devices.

FDA and the National Institutes of Health jointly published mock presubmission reviews of two examples of multiplex proteomic tests, one a mass spectrometry-based test and one protein array-based test. This publication provides feedback for product developers on how multiplex biomarker tests might be viewed by the agency [7]. This publication also demonstrates some of the unique analytical challenges (such as cross-reactivity) and clinical challenges (consideration of how the multiplex data would be interpreted by the clinician) that need to be considered.

PSA and CA-125 are illustrative examples of how clinical studies are typically targeted for intended use. For example, the most recent PMA approval of a PSA device for diagnosis of prostate cancer (Access® Hybritech® p2PSA), the sponsor performed a clinical study using a 658-member patient cohort of mostly prospectively enrolled subjects, both with and without prostate cancer. All patients were subjected to biopsy, the gold standard diagnostic method, to determine the clinical truth, or reference diagnosis.

In contrast, CA-125 devices are intended for the monitoring of ovarian cancer and response to therapy (class II claim). For the most recent 510(k) clearance of a CA-125 device (Dimension Vista® LOCI CA 125 Flex® Reagent Cartridge), the sponsor performed a retrospective study on 330 frozen specimens taken from 75 subjects during follow-up surveillance for ovarian cancer progression [86]. The change in CA-125 value, for both test and predicate device, was compared to the gold standard diagnosis (whether the patient’s disease status was classified as progression or no progression), and then the performance of the test device was compared to the performance of the predicate device to determine whether the two devices were substantially equivalent.

### Approval process workflow

The approval process for a novel protein biomarker typically starts with the sponsor preparing a presubmission application to the agency. The presubmission (formerly called pre-IDE) generally includes a statement of the assay’s intended use, a description of the technology, and a proposal for analytical and clinical studies [87]. The sponsor will typically provide a list of questions for FDA. Feedback is available to the sponsor either by written feedback, or feedback through a teleconference or a face-to-face meeting between the sponsor and the agency. During the presubmission process, the FDA and sponsor teams will usually come to an agreement whether the biomarker assay device will require a PMA, 510(k), or de novo submission. The sponsor will then perform the necessary analytical and clinical studies.

Significant risk clinical studies need to be reported to FDA under an Investigational Device Exemption (IDE) application (21 CFR 812). A clinical study for a protein biomarker assay would be considered significant risk if a) the data were reported to patients and used for patient care, or b) if the study involved an invasive sampling procedure, such as a biopsy. Serum or plasma sampling from patients is not considered invasive. For many studies involving biomarker assay, an IDE is not required because the data from biomarker assay studies can be compared to a gold standard method which is used for patient care. For example, a clinical study of a potential serum-based lung cancer protein biomarker can include comparison to computed tomography (CT) screening and the CT screening results can be used for patient care while the biomarker results are not reported to the patients. Such a study would likely not require an IDE.

The sponsor then submits a 510(k) or PMA application, along with the appropriate user fees, to the agency. The agency is responsible for reviewing the submission within time frames specified by the 2012 FDASIA user fee agreement – 90 days for a 510(k) and 180 days for a PMA. If FDA finds deficiencies, the agency will send the sponsor a list of these deficiencies and the submission is put on hold while the sponsor addresses the agency’s requests. The sponsor and reviewer can work together to solve any further issues that remain after the hold has been lifted.

If a Class II device is found to be substantially equivalent and cleared it may be legally marketed in the United States. FDA also determines the complexity level of the test, to determine in which type of CLIA-certified laboratories the test may be performed. Tests that are meant to be run at home, or in non-CLIA certified environments such as a physician’s office, or other point-of-care settings, have specialized review requirements.

For a PMA submission, the FDA determines whether the device is safe and effective, based on the data provided by the sponsor. A premarket inspection of the firm is also required, during which FDA determines if the firm’s manufacturing processes are in accordance with the quality system for medical devices (21 CFR 820). Firms with devices approved under a PMA also have significant postmarket responsibilities – all manufacturing or design changes must be reported to FDA on an annual basis, and any manufacturing or design change that potentially may affect the safety and effectiveness of the device requires the submission of a PMA supplement application.

## Conclusions

In this review, we discussed the issues and challenges for the translation of proteomic biomarkers into FDA approved cancer diagnostics. To be successful, one should develop a roadmap and identify the key steps that are critical in this process. We emphasized the importance of assay development and defined the criteria for both analytical and clinical performances. Understanding the FDA approval process is important for the development of any clinical diagnostics. Depending on the specific intended use, one could follow either the 510 K or the PMA route. Ultimately, it is the test (device) for the cancer biomarker that will be evaluated and approved by the FDA for clinical use. We understand that obtaining FDA approval for tumor markers does not imply automatic clinical acceptance by clinicians. It is important to demonstrate strong clinical utilities with publication in respectable scientific journals. In addition, these FDA approved tumor markers should be incorporated into the practice guideline established by clinical societies. While we recognize that the road from biomarker discovery, validation, and regulatory (FDA) approval to the translation into the clinical setting could be long and difficult, however, the reward for patients, clinicians and scientists could be rather significant.

## Abbreviations

FDA: Food and drug administration; SD: Standard deviation; CV: Coefficient of variation; hCG: Human choriogonadotropin; PSA: Prostate specific antigen; LoD: Limit of detection; LoB: Limit of blank; LoQ: Limit of quantitation; AFP: Alpha-fetoprotein; CEA: Carcinoembryonic antigen; ROC: Receiver operating characteristic; AUC: Area under the curve; PPV: Positive predictive value; NPV: Negative predictive value; CA-125: Cancer antigen 125; CDRH: Center for devices and radiological health; PMA: Premarket approval application; IDE: Investigational device exemption; CT: Computed tomography.

## Competing interests

The authors declare that they have no competing interests.

## Authors’ contributions

All authors were involved in the drafting and revising of the manuscript. All authors read and approved the final manuscript.
